# Fostering links, building trust, and facilitating change: connectivity helps sustain longitudinal integrated clerkships in small rural and remote communities

**DOI:** 10.1186/s12909-024-06373-3

**Published:** 2024-11-29

**Authors:** Brendan Carrigan, William MacAskill, Janani Pinidiyapathirage, Sherrilyn Walters, Lara Fuller, Kay Brumpton

**Affiliations:** 1 Rural Medical Education Australia, Toowoomba, Australia; 2 Rural Clinical School, Griffith University, Toowoomba, Australia; 3 Rural Community Clinical School, Deakin University, Geelong, Australia

**Keywords:** Longitudinal integrated clerkship, Rural health, Medical education, Health workforce, Connectivity

## Abstract

**Background:**

Maldistribution of medical professionals presents a significant challenge globally and leads to inequitable healthcare access, particularly in remote areas. Longitudinal integrated clerkships (LICs) in rural areas can improve workforce distribution and may be an innovative contributor to solving maldistribution issues. However, to align with healthcare needs, LICs must be sustainable in small communities, which often have a limited medical workforce. This study investigates the key elements underpinning LIC sustainability in small communities.

**Methods:**

This study adopted a constructivist research paradigm in which participants’ constructions of their experiences supporting LICs in small rural communities were explored. Participants were conveniently sampled from the LIC community of practice attending the 2021 virtual annual conference of the Consortium of Longitudinal Integrated Clerkships. Data were collected through video recording and thematically analysed to identify barriers and enablers to running sustainable LIC programmes.

**Results:**

Eleven participants fulfilling key roles within LICs, including clinical school directors, program coordinators, and clinical educators, were recruited for the study. Thematic analysis indicated that it is Connectivity, expressed through three subthemes, Fostering Links, Building Trust, and Facilitating Change, which underpins sustainable LICs in small communities.

**Conclusions:**

Connectivity is a strong mediator for sustainability of LICs and may be the central defining theme of LICs. Increasing connectivity through prioritizing community engagement, trust-building, and strategic investment enhances the sustainability of rural LICs, ensuring their continued positive contribution to medical workforce distribution in underserved areas.

## Background

The maldistribution of the medical workforce, where medical workers are concentrated in densely populated metropolitan areas at the expense of increasingly remote regions, is a persistent global problem [1]. The shortage of health professionals in rural localities is multidisciplinary, impacting all fields and specialties [2]. With low population, workforce pressures are intensified, and the shortfall leads to inequitable health care access for rural and remote populations [1, 3, 4]. Therefore, innovative ‘place-based’ solutions that align workforce distribution with local healthcare needs are required.

Rural longitudinal integrated clerkships (LICs) have an established record in improving medical workforce maldistribution [5, 6]. LICs differ in context and structure from traditional block rotation medical programs [7]. Students are immersed in generalist clinical settings, providing comprehensive patient care with the aim of learning whole of curriculum in an integrated manner [8, 9]. The benefits of LICs are well established and include improvements in clinical skills, high-level cognition, patient-centred communication, culturally safe practice, and student satisfaction [5, 9–11]. Further, the longer immersive placement within rural communities fosters a sense of belonging and kinship through repeated clinical and social interactions [12, 13]. This engagement and relationship building across the learning environment, professional interactions, and integration within the local community has been described as “connectivity” [13]. Connectivity not only enhances student experience but is also an important driver of attraction to rural programs and the retention of students or medical professionals in rural practice [13–16].

Despite their established benefits, supporting LICs in smaller or remote regions is challenging. Students report suffering isolation from existing social networks and associated anxiety [17–19]. Areas of workforce pressure, where LICs are often located, suffer greater staff turnover, which threatens the sustainability of teaching faculty and disrupts the clinician‒student relationship central to LICs [20, 21]. In smaller teams, relationship tensions and interpersonal conflicts have greater potential to disrupt the clerkship experience [21, 22]. Infrastructure needs, such as accommodation or technological requirements, and the need to travel may increase costs [20–22]. These factors are likely further exacerbated in small communities; however, these communities experience the most severe workforce shortages, which LICs are proposed to alleviate [3, 9].

There is a paucity of literature describing what promotes the suitability of LICs for rural contexts [20]. By examining the knowledge and experience of medical education experts who develop and deliver LICs, this paper investigates what factors promote the sustainability of LICs in “small communities”. Understanding what makes LICs sustainable in these communities may assist with more widespread adoption of this model to address workforce maldistribution, improve workforce retention, enhance healthcare accessibility, and support the provision of high-quality medical student training aligned with community healthcare needs.

## Materials and methods

### Study setting and context

This study adopted a constructivist research paradigm in which participants’ constructions of their experience supporting LICs in small rural communities were explored [23]. Participants were conveniently sampled from the LIC community of practice attending the virtual annual conference of the Consortium of Longitudinal Integrated Clerkships (CLIC) in 2021. CLIC brings together a global network of medical educational experts who develop and deliver LICs [24].

This paper defines a community as a group of people living in the same region or town. The perception of what makes a community “small” varies by context. The research was generated in the context of an LIC, the ‘Longlook’ program, which operates in a regional center as well as large and medium rural towns (Modified Monash categories 2–4) [15, 25]. Student placement capacity within communities with procedural birthing hospitals is approaching saturation. To expand, additional placements will need to occur in communities without procedural birthing services, which we define as “small communities”. These communities have limited access to healthcare providers (i.e., a small community of practice) or are in areas of low population density.

### Participants and data collection

The participants were attendees in a sixty-minute CLIC conference virtual session entitled “How small is too small?”. Prior to commencement, the study aims were outlined, and attendees were offered the opportunity to be involved in the study. All attendees consented to participate in the study and for the online session to be video recorded. Participants were part of the LIC community of practice and were therefore opportunely placed to contribute to developing knowledge of rural and remote clinical education [26, 27].

The participants were asked to consider factors that sustain LIC placements in small rural communities. An initial five-minute presentation led by the presenting team (BC, KB) introduced and framed the conversation topics and was followed by group discussion facilitated by LF. On the basis of the experience of the authors in supporting LICs, four questions were developed to prompt discussion by the participants:

1. What is a small community for a LIC?
2. What are the main barriers to LICs in small communities?
3. How do we best support students in LICs in small communities?
4. What other factors ensure the success of LICs in small communities?

To conclude, the key ideas discussed were summarised and presented back to participants by BC to ensure that their perspectives were captured and understood.

### Data analysis

Session recordings were transcribed using SONIX™ (Sonix.ai, San Francisco, USA) transcription software. The transcripts were manually checked for accuracy and deidentified by SW prior to distribution within the research team. Participants were assigned a participant number for analysis and reporting.

Previously described methods of thematic analysis and synthesis were used to analyse the data [28]. NVivo (QSR International, VIC, Australia) qualitative data management software was used for data management during analysis. Transcripts were initially independently coded by JP and SW. Code books were compared and discussed to develop a final codebook used for line-by-line coding. Data were synthesized by JP and SW to identify broad salient themes. Themes were validated by BC as to their relevance and applicability to the research questions prior to discussion with the broader research team. Analysis and themes were circulated to participants for comment prior to manuscript submission.

## Results

Eleven participants attended the session and were representative of the following countries: Australia, South Africa, the United Kingdom, and the United States. Participant roles within LICs included clinical school directors, program coordinators, and clinical educators.

Thematic analysis indicated that it is Connectivity, expressed through three sub-themes: Fostering Links, Building Trust, and Facilitating Change, which underpins sustainable LICs in small communities. These are described separately below.

### Fostering links

Relationship building is central to establishing and maintaining connectivity. Participants discussed the importance of investing in relationships through (1) creating and maintaining LIC–community relationships, (2) emphasizing interprofessional learning, and (3) maintaining peer–peer connections.

“You can’t underestimate the value of putting investment into the relationships… you need the infrastructure… but at the end of the day, I actually think it’s about the relationships.” P11 Clinical Educator

The central relationships supporting LICs were those between the LIC and the local community. Relationship building with community representatives was seen as a key step in ensuring the success of LIC programs. Fostering mutual ownership of the LIC program by the local community was seen as a crucial component of building the LIC-community relationship.

“It’s that reciprocal relationship building. So that there’s mutual ownership of this LIC and this journey… we need to factor in the time that it takes to build this relationship because [The university is] coming in as a new partner, and we need to take the time to build it, and to nurture it…” P3 Clinical Educator

Pivotal in building this relationship is a “community broker”, a person embedded within the community who is the conduit with the LIC program. This person is tasked with ensuring that students are accepted into the local area and that communities are valued by the students and the university.

“Making sure that there’s a dedicated person whose job is… a broker. So, brokering in a number of ways with the health service in terms of actually achieving the placement… acting as kind of like the conduit so… that small place actually feels connected, supported, that they feel part of the university, but also that the students feel part of the community” P11 Clinical Educator

Participants discussed the need to focus on investing in interprofessional relationships to facilitate efficiencies within the small community of practice, promoting both vertical and horizontal learning.

“The interprofessional stuff… education that’s happening for other learners… because traditionally [different professions have] been very separate. [Education] is happening here for the nursing students, and they’ve probably got a support person and then the medical students are over here… it’s trying to bring those two groups together… and be more efficient.” P1 Clinical School Director

Participants also discussed the importance of creating and maintaining peer–peer connections and supports. Students within a small town can feel vulnerable, and connection to peers across the LIC can help alleviate this.

“Some of our sites… only have two students, [we make] sure that the students are in regular contact with the rest of the LIC students… The students are given access to the university’s [online conferencing platform]… with [virtual] lounge rooms… so that they can have a student network across not just the small town, but across all the small towns.” P11 Clinical Educator

“We try to… bring our students together… They discuss their [patient cohort]… they get to see their peers… they’re starting to slowly develop a bond with some of their LIC peers.” P5 Clinical Educator

### Building trust

Connectivity requires trust to build and strengthen the relationships between stakeholders. Stable delivery of clinical services; alignment of curriculum and clinical practice; and the delivery on promises made by program directors, educators, and universities affect student and community trust in LIC programs. Students, the university, and the community need to trust that the curriculum is achievable in the LIC.

“When I look at a town and think, could we put a student here? It comes back to the curriculum and the learning objectives that we need to meet and can this town tick those boxes.” P1 Clinical School Director

Achieving curriculum delivery requires a flexible approach, as each community has distinctive service needs and capabilities. This at times led to situations where creative solutions were required to ensure curriculum delivery.

“Flexibility in terms of curriculum and assessment and program design, that as long as we’re achieving the same outcomes, that we need to be flexible in how that happens… that includes being responsive to individual needs. And that may be different for different areas.” P6 Program Coordinator

“Do it based on student need… You’re not seeing any surgery, okay? We’ll get you up to the nearest town to do some surgery or women’s health to go and, labour wards a big one for the smaller towns to get obstetric experience.” P1 Clinical School Director

Service stability, the continued availability of suitable healthcare staff and their ability to provide health services to the community affect a community’s confidence that the LIC is deliverable. Small services are particularly vulnerable to the instability of staffing.

“What you worry about is, you know that [the health service is] right on the edge. If something happens to one of the doctors, that’s your GP [supervisor]. It’s very vulnerable.” P11 Clinical Educator

Programs can work to enhance this stability by investing resources that capacitate both education and service delivery.

“We really wanted to… branch out and build capacity, we’ve employed through providing a fraction [of a full-time employee] to the health service… a nurse educator within the health service.” P1 Clinical School Director

Most important however was that programs were accountable to the community in which they were placed and delivered on the promises they made. If trust is granted, it should not be broken, as it takes time to rebuild this relationship.

“We have history of people going into communities and promising things, and then it doesn’t happen and it makes it really harder for the next person to enter.” P3 Clinical Educator

### Facilitating change

Participants explored the importance of building the educational capacity of local sites through supervisor training and support and establishing and supporting infrastructure. Relationships require investment, and to create change to promote connectivity, programs need to invest in capacity building. This included improving physical infrastructure and supporting student travel.

“You need things like accommodation, travel support, communication support. Actually, one thing that’s been really powerful in [our region] is bringing telecommunications infrastructure with us and supporting the local [partner’s] negotiations with the [telecommunication network] providers in terms of beefing up infrastructure.” P11 Clinical Educator

Supervisors were seen as key assets in the success of an LIC, and programs need to invest in supervisor development and build educational capacity.

“Supporting [supervisors]…. We’ve run a series of faculty development [activities] across our network… the rural school funded [supervisors] to [travel]… to meet with… other staff… other people with similar roles. [Supervisors] get to be involved in conversations around the work that they’re doing [and] learning a bit as they go.” P11 Clinical Educator

## Discussion

Here, we present key principles fostering community links, building and maintaining community trust in the LIC program, and investing in the community to facilitate change to sustain LICs within small communities. These principles indicate that to promote the sustainability of LICs in small communities, programs need to enhance connectivity, which has been identified as the overarching theme in this analysis. Although originally described in reference to student learning within an LIC, our study suggests that the influence of connectivity is broader and may be the central defining theme of sustainable rural LICs [13, 15].

Within a rural LIC, Roberts et al. [13] describe connectivity as a powerful mediator of authentic learning within LICs. Connectivity promotes relationships both within and between professions, enhancing learning opportunities, particularly through informal opportunities [13]. Agentic engagement with the broader community affords a deeper social economic view, promotes deeper integration within the community, and improves the interactions between students and patients [13, 29]. Connectivity is valued by students within LICs, where programs enhance connectivity students are more likely to remain in rural medical programs [15]. Further this engagement, particularly early in career, has a strong influence on progression to independent rural practice [30, 31]. Our study demonstrates that connectivity is also a strong mediator of the sustainability of LICs within small communities.

Central to promoting connectivity is what our participants label a ‘community broker’, a member of the facility tasked with ensuring the transition and integration of students within the professional and social community. Within LICs, this role is described by various names. Pinidiyapathirage et al. [16] describe champions who develop and sustain relationships with key stakeholders to facilitate LIC placements. Green et al. [32] report the importance of a ‘clinical coordinator’ who maintains communication and relationships between key stakeholders such as students and the community. Bartlett et al. [20] highlight that leaders of rural medical programs must lead and facilitate community engagement to be “visible, accessible, responsive, and adaptable to changing local contexts”. Strasser et al. [33] describe the importance of both community-engaged medical education and community mentors, who provide students with community orientation and insights to facilitate engagement. This role is not necessarily separate from that of the clinical supervisor, with LIC supervisors actively facilitating access to noncurricular community activities to promote student learning and engagement [34]. Regardless of who performs these roles, they are central to the connectivity that promotes LIC sustainability.

Educators also describe value in facilitating peer connectivity to alleviate the effects of social isolation and student anxiety described elsewhere [17–19]. Students highly value peer connectivity: it is effective for promotion of LICs to other students and is a strong mediator of student retention within LICs [15, 16]. Peer connectivity can be fostered through multiple means, including simply placing students in groups to allow direct peer support [19, 20]. Formal near-peer support helps the transition of new students into the often initially disorientating LIC learning setting [35]. Broadly, continuity of peer groups provides guidance about expectations, ways of interacting, and implicit rules of the clinical setting to incoming students [36].

Fostering connectivity requires building community trust in the LIC. Our participants discussed the importance of a flexible curriculum that can adapt to meet the needs of their community. Worley [37] proposes a symbiotic curriculum where students sit at the centre of the clinical, institutional, social, and personal axes. The relationships across these axes matter, and the key to achieving a high-quality, sustainable education program is to ensure that these relationships are mutually beneficial [37, 38]. Sustainability requires flexibility to allow a program to be adapted, which is dependent on the community’s characteristics and strengths [18, 19, 39]. LICs are inherently responsive and adaptable, allowing stronger community integration and shared ownership, which promotes connectivity [20].

Finally, it is important to invest in the community’s resources and relationships to foster connectivity and support sustainability. For LICs to succeed in small communities, our participants highlighted the need to invest in infrastructure, such as information technology and accommodation, and supervision capacity. Multiple authors have discussed the need for adequate preexisting infrastructure [18–20, 38, 40]; however, for small communities, LIC programs may need to actively fund this supporting infrastructure.

This study is limited by its sample size, session length and reliance on self-selection. Additionally, the CLIC conference session was delivered virtually across multiple time zones, which may have impaired engagement in some regions. Although the participants had rich and diverse experiences, they may not have described the LIC experience completely. Further research to confirm these findings with a larger diverse sample from multiple contexts is warranted.

## Conclusions

If LICs are a viable solution for improving medical workforce maldistribution, they must be sustainable in small rural communities. This study examined the experiences within the LIC community of practice to identify barriers and enablers to this aim. Our findings indicate that enhancing connectivity through fostering links, building trust, and facilitating change is key to sustainable LICs in small communities. When students are integrated within community networks, programs can create tangible improvements on the ground and deliver achievable and flexible medical education programs responsive to community needs.

## Data Availability

The deidentified transcriptions are available from the corresponding author upon reasonable request.

## Abbreviations

LIC: Longitudinal integrated clerkship
CLIC: Consortium of Longitudinal Integrated Clerkships

## References

[1] World Health Organization. WHO guideline on health workforce development, attraction, recruitment and retention in rural and remote areas. Geneva: World Health Organization; 2021 2021. 34057827

[2] GBD 2019 Human Resources for Health Collaborators. Measuring the availability of human resources for health and its relationship to universal health coverage for 204 countries and territories from 1990 to 2019: a systematic analysis for the global burden of Disease Study 2019. Lancet. 2022;399(10341):2129–54. 35617980 10.1016/S0140-6736(22)00532-3 PMC9168805

[3] Australian Institute of Health Welfare. Health workforce. Canberra: AIHW; 2022.

[4] Australian Institute of Health Welfare. Rural and remote health. Canberra: AIHW; 2023.

[5] Walters L, Greenhill J, Richards J, Ward H, Campbell N, Ash J, et al. Outcomes of longitudinal integrated clinical placements for students, clinicians and society. Med Educ. 2012;46(11):1028–41. 23078680 10.1111/j.1365-2923.2012.04331.x

[6] Beattie J, Binder M, Fuller L. Rural longitudinal integrated clerkships and medical workforce outcomes: a scoping review. Med Teach. 2024;46(4):545–55. 37769044 10.1080/0142159X.2023.2260082

[7] Fuller L, Lawson M, Beattie J. The impact of clerkship model and clinical setting on medical student’s participation in the clinical workplace: a comparison of rural LIC and rural block rotation experience. Med Teach. 2021;43(3):307–13. 33307934 10.1080/0142159X.2020.1839032

[8] Norris TE, Schaad DC, DeWitt D, Ogur B, Hunt DD, members of the Consortium of Longitudinal Integrated Clerkships. Longitudinal integrated clerkships for medical students: an innovation adopted by medical schools in Australia, Canada, South Africa, and the United States. Acad Med. 2009;84(7):902–7. 19550184 10.1097/ACM.0b013e3181a85776

[9] Worley P, Couper I, Strasser R, Graves L, Cummings BA, Woodman R, et al. A typology of longitudinal integrated clerkships. Med Educ. 2016;50(9):922–32. 27562892 10.1111/medu.13084

[10] Purea P, Brumpton K, Kumar K, Pinidiyapathirage J. Exploring the learning environment afforded by an Aboriginal Community Controlled Health service in a rural longitudinal integrated clerkship. Educ Prim Care. 2022;33(4):214–20. 35343387 10.1080/14739879.2022.2054371

[11] Teherani A, Irby DM, Loeser H. Outcomes of different clerkship models: longitudinal integrated, hybrid, and block. Acad Med. 2013;88(1):35–43. 23165275 10.1097/ACM.0b013e318276ca9b

[12] Daly M, Roberts C, Kumar K, Perkins D. Longitudinal integrated rural placements: a social learning systems perspective. Med Educ. 2013;47(4):352–61. 23488755 10.1111/medu.12097

[13] Roberts C, Daly M, Held F, Lyle D. Social learning in a longitudinal integrated clinical placement. Adv Health Sci Educ Theory Pract. 2017;22(4):1011–29. 27915432 10.1007/s10459-016-9740-3

[14] Campbell N, McAllister L, Eley D. The influence of motivation in recruitment and retention of rural and remote allied health professionals: a literature review. Rural Remote Health. 2012;12:1900. 22845190

[15] Carrigan B, Bass L, Pinidiyapathirage J, Walters S, Woodall H, Brumpton K. Connectivity is the key to longer rural placement: retaining students on rural longitudinal integrated clerkships. Med Teach. 2024;46(2):225–31. 37557884 10.1080/0142159X.2023.2243025

[16] Pinidiyapathirage J, Heffernan R, Carrigan B, Walters S, Fuller L, Brumpton K. Recruiting students to rural longitudinal integrated clerkships: a qualitative study of medical educationists’ experiences across continents. BMC Med Educ. 2023;23(1):974. 38115001 10.1186/s12909-023-04949-z PMC10731800

[17] Hense H, Harst L, Kuster D, Walther F, Schmitt J. Implementing longitudinal integrated curricula: systematic review of barriers and facilitators. Med Educ. 2021;55(5):558–73. 33099784 10.1111/medu.14401

[18] Bartlett M, Dowell J, Graham F, Knight K, Law S, Lockwood P, et al. Dundee’s Longitudinal Integrated Clerkship: drivers, implementation and early evaluation. Educ Prim Care. 2019;30(2):72–9. 30652938 10.1080/14739879.2018.1564889

[19] Couper I, Worley PS, Strasser R. Rural longitudinal integrated clerkships: lessons from two programs on different continents. Rural Remote Health. 2011;11(2):1665. 21449620

[20] Bartlett M, Couper I, Poncelet A, Worley P. The do’s, don’ts and don’t knows of establishing a sustainable longitudinal integrated clerkship. Perspect Med Educ. 2020;9(1):5–19. 31953655 10.1007/s40037-019-00558-z PMC7012799

[21] Brown ME, Anderson K, Finn GM. A narrative literature review considering the development and implementation of Longitudinal Integrated clerkships, including a practical guide for application. J Med Educ Curric Dev. 2019;6:2382120519849409. 31206031 10.1177/2382120519849409 PMC6537286

[22] Ellaway R, Graves L, Berry S, Myhre D, Cummings BA, Konkin J. Twelve tips for designing and running longitudinal integrated clerkships. Med Teach. 2013;35(12):989–95. 23883396 10.3109/0142159X.2013.818110 PMC3836395

[23] Shannon-Baker P. Philosophical underpinnings of mixed methods research in education. In: Tierney RJ, Rizvi F, Ercikan K, editors. International Encyclopedia of Education (Fourth Edition). Oxford: Elsevier; 2023. pp. 380–9.

[24] The Consortium of Longitudinal Integrated Clerkships. About 2022. https://clicmeded.com/about/. Accessed 25 Sept 2022.

[25] Australian Government Department of Health. Modified Monash Model 2020. https://www.health.gov.au/topics/rural-health-workforce/classifications/mmm. Accessed 12 Feb 2024.

[26] Walton E, Carrington S, Saggers B, Edwards C, Kimani W. What matters in learning communities for inclusive education: a cross-case analysis. Prof Dev Educ. 2019;48(5):1–15.

[27] Vescio V, Ross D, Adams A. A review of research on the impact of professional learning communities on teaching practice and student learning. Teach Teach Educ. 2008;24(1):80–91.

[28] Braun V, Clarke V, Thematic Analysis. A Practical Guide: SAGE; 2021.

[29] Reeve J, Tseng C-M. Agency as a fourth aspect of students’ engagement during learning activities. Contemp Educ Psychol. 2011;36(4):257–67.

[30] McGrail MR, Doyle Z, Fuller L, Gupta TS, Shires L, Walters L. The pathway to more rural doctors: the role of universities. Med J Aust. 2023;219(Suppl 3):S8–13. 37544002 10.5694/mja2.52021

[31] O’Sullivan BG, McGrail MR, Russell D, Chambers H, Major L. A review of characteristics and outcomes of Australia’s undergraduate medical education rural immersion programs. Hum Resour Health. 2018;16(1):8. 29386024 10.1186/s12960-018-0271-2 PMC5793366

[32] Green E, Quilliam C, Sheepway L, Hays CA, Moore L, Rasiah RL, et al. Identifying features of quality in rural placements for health students: scoping review. BMJ Open. 2022;12(4):e057074. 10.1136/bmjopen-2021-057074 PMC8995951 35396299

[33] Strasser R, Worley P, Cristobal F, Marsh DC, Berry S, Strasser S, et al. Putting communities in the driver’s seat: the realities of community-engaged medical education. Acad Med. 2015;90(11):1466–70. 26017354 10.1097/ACM.0000000000000765

[34] Gupta S, Howden S. Medical students’ perceptions of ‘community’ in a longitudinal integrated clerkship. Educ Prim Care. 2021;32(3):157–65. 33342354 10.1080/14739879.2020.1850211

[35] Illing JC, Crampton PE. Collaborative relationships and learning in rural communities. Med Educ. 2015;49(9):852–4. 26296398 10.1111/medu.12784

[36] Chou CL, Teherani A, Masters DE, Vener M, Wamsley M, Poncelet A. Workplace learning through peer groups in medical school clerkships. Med Educ Online. 2014;19:25809. 25427851 10.3402/meo.v19.25809 PMC4245452

[37] Worley P. Relationships: a new way to analyse community-based medical education? (part one). Educ Health (Abingdon). 2002;15(2):117–28. 14741960 10.1080/13576280210133062

[38] Prideaux D, Worley P, Bligh J. Symbiosis: a new model for clinical education. Clin Teach. 2007;4(4):209–12.

[39] Poncelet A, Bokser S, Calton B, Hauer KE, Kirsch H, Jones T et al. Development of a longitudinal integrated clerkship at an academic medical center. Med Educ Online. 2011;16. 10.3402/meo.v16i0.5939 PMC3071870 21475642

[40] Poncelet AN, Mazotti LA, Blumberg B, Wamsley MA, Grennan T, Shore WB. Creating a longitudinal integrated clerkship with mutual benefits for an academic medical center and a community health system. Perm J. 2014;18(2):50–6. 10.7812/TPP/13-137 PMC4022558 24867551

